# The development of proximity labeling technology and its applications in mammals, plants, and microorganisms

**DOI:** 10.1186/s12964-023-01310-1

**Published:** 2023-09-30

**Authors:** Jieyu Guo, Shuang Guo, Siao Lu, Jun Gong, Long Wang, Liqiong Ding, Qingjie Chen, Wu Liu

**Affiliations:** 1 School of Basic Medical Sciences, Xianning Medical College, Hubei University of Science and Technology, Xianning, Hubei 437000 China; 2 School of Pharmacy, Xianning Medical College, Hubei University of Science and Technology, Xianning, Hubei 437000 China; 3 Medicine Research Institute, Hubei Key Laboratory of Diabetes and Angiopathy, Xianning Medical College, Hubei University of Science and Technology, Xianning, Hubei 437000 China

**Keywords:** Proximity labeling, APEX, BioID, TurboID, miniTurbo

## Abstract

Protein‒protein, protein‒RNA, and protein‒DNA interaction networks form the basis of cellular regulation and signal transduction, making it crucial to explore these interaction networks to understand complex biological processes. Traditional methods such as affinity purification and yeast two-hybrid assays have been shown to have limitations, as they can only isolate high-affinity molecular interactions under nonphysiological conditions or in vitro. Moreover, these methods have shortcomings for organelle isolation and protein subcellular localization. To address these issues, proximity labeling techniques have been developed. This technology not only overcomes the limitations of traditional methods but also offers unique advantages in studying protein spatial characteristics and molecular interactions within living cells. Currently, this technique not only is indispensable in research on mammalian nucleoprotein interactions but also provides a reliable approach for studying nonmammalian cells, such as plants, parasites and viruses. Given these advantages, this article provides a detailed introduction to the principles of proximity labeling techniques and the development of labeling enzymes. The focus is on summarizing the recent applications of TurboID and miniTurbo in mammals, plants, and microorganisms.

Video Abstract

**Supplementary Information:**

The online version contains supplementary material available at 10.1186/s12964-023-01310-1.

Molecular interactions are the foundation of life processes, including protein-protein interactions (PPIs), protein-RNA interactions, and protein-DNA interactions, which are closely regulated by proteins, nucleic acids, and their interactions. As molecular interactions lie at the core of most biological processes, elucidating the network of interactions between molecules is not only crucial for analyzing protein abundance and localization, PPIs, and interactions between proteins and DNA/RNA but also enhances our understanding of cell growth, differentiation, and apoptosis processes [1]. Traditional methods for studying molecular interactions include affinity purification, the yeast two-hybrid system [2, 3], the bimolecular fluorescence complementation assay (BiFC) [4–6], the glutathione S-transferase pull-down assay (GST pull-down assay) [7, 8], and coimmunoprecipitation (Co-IP) [9]. Affinity purification involves using antibodies to enrich and identify molecules stably interacting with the target protein through affinity purification and mass spectrometry, deepening our understanding of protein interaction networks in microorganisms, plants, and mammals [10]. Moreover, affinity purification can be combined with cross-linking and nucleic acid sequencing to explore protein-nucleic acid interactions, such as chromatin immunoprecipitation sequencing (ChIP-seq) [11] and RNA immunoprecipitation sequencing (RIP-seq) [12]. However, affinity purification often loses weak or transient interactions during cell lysis and washing, and this method has difficulty capturing insoluble targets, such as chromatin and membrane-associated proteins, or proteins lacking high-affinity antibodies as bait. The yeast two-hybrid system is another method for exploring protein‒protein, protein‒RNA, and protein‒DNA interaction networks in living cells [13]. Its principle is based on the fact that transcription of the yeast cell’s reporter gene requires the participation of transcriptional activators. Therefore, this method is sensitive to weak or transient protein interactions and can screen for thousands to millions of potential molecular interactions. Although this method is simple and efficient, traditional yeast two-hybrid technology can only detect interactions within the nucleus and has drawbacks such as low transformation efficiency and frequent false-positive detection results [14]. BiFC is based on in vivo recombination of fluorescent proteins, and its signals are easily quantified, allowing efficient cell-based high-throughput screening. However, BiFC complexes usually become stable after binding to fluorescent protein fragments, and thus temporal changes in protein interactions cannot be monitored in real time [4].

To address these limitations, proximity labeling (PL) technology has been introduced into proteomics [15]. This technique can replace methods such as immunoprecipitation and biochemical analysis to study large molecular complexes, organelles, and protein interaction networks [16, 17]. The principle of proximity labeling technology involves fusing a genetically encoded biotin ligase to specific proteins or subcellular regions (such as synaptic clefts, mitochondrial intermembrane spaces, various membrane-less organelles, and organelle contact sites) [18–21]. This targets the enzyme to the desired protein complex or organelle. Subsequently, a small-molecule substrate, such as biotin, is added, which initiates the covalent labeling of endogenous proteins within nanometer proximity of the enzyme. The labeled target protein complex or organelle can be affinity purified and enriched using streptavidin-coated magnetic beads, and the results can be analyzed through mass spectrometry or high-throughput sequencing [22].

 In recent years, proximity labeling technology has rapidly developed and can be classified into two categories based on the labeling enzymes: peroxidases and biotin ligases [23–26]. Peroxidases include ascorbate peroxidase (APEX) and APEX2, but biotin ligases are more widely used. The latter category includes BioID, BioID2, BASU, TurboID, miniTurbo, Split-BioID, and Split-TurboID, among others. This review summarizes the characteristics of these neighboring labeling technologies in Table 1.

**Table 1:**
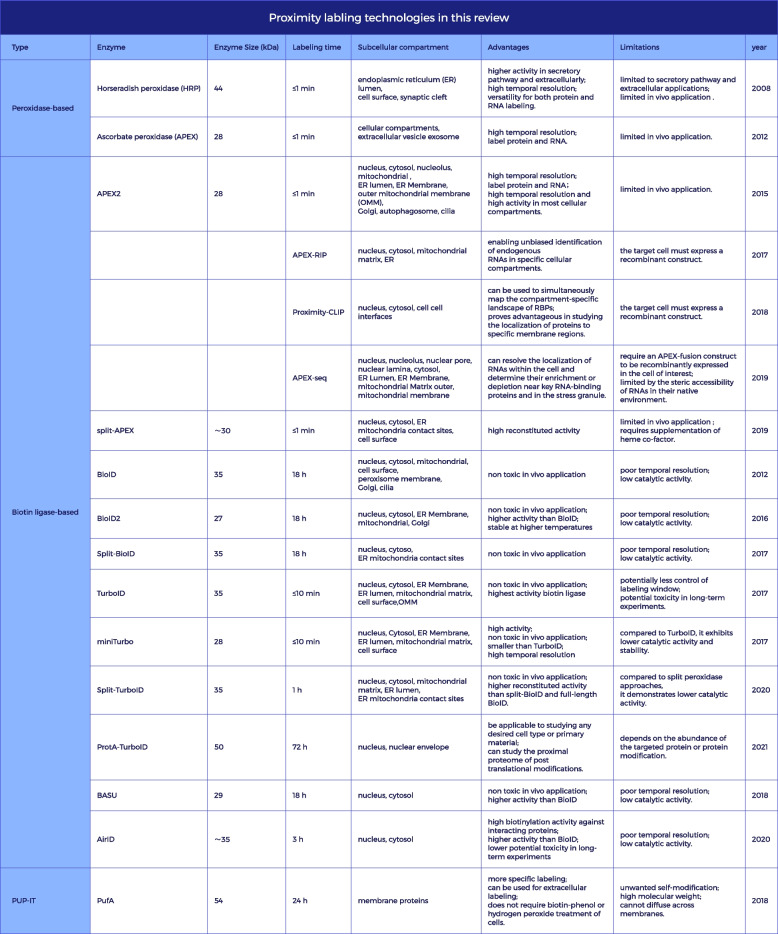
Proximity labling technologies in this review

Although there are many proximity labeling enzymes, they share the following characteristics: First, the enzyme is fused with the target protein and expressed in living cells without disrupting the localization, function, and interactions of the target protein. Second, the enzyme is fused with a signal peptide and targeted to specific subcellular compartments. After the enzyme substrate is added to the cells, the enzyme reacts with the substrate to produce a reactive intermediate that covalently labels nucleic acids or proteins in proximity [27]. To date, proximity labeling technology has been applied in various biological fields, including exploring low-affinity and insoluble proteins, segmenting proteins to study organelles, labeling weak and transiently interacting proteins to understand the biological properties of membrane proteins, and amplifying local signals for immunofluorescently labeling cell structures [28, 29]. Disruptions in molecular interaction networks are closely related to human diseases, including immune disorders, neurodegenerative diseases, and cancer [30–33]. Therefore, studying molecular interactions is of great importance. This article will focus on introducing the development, principles, and applications of proximity labeling technology.

## Development and principles of proximity labeling technology

### Proximity labeling technology based on peroxidases

**Fig. 1:**
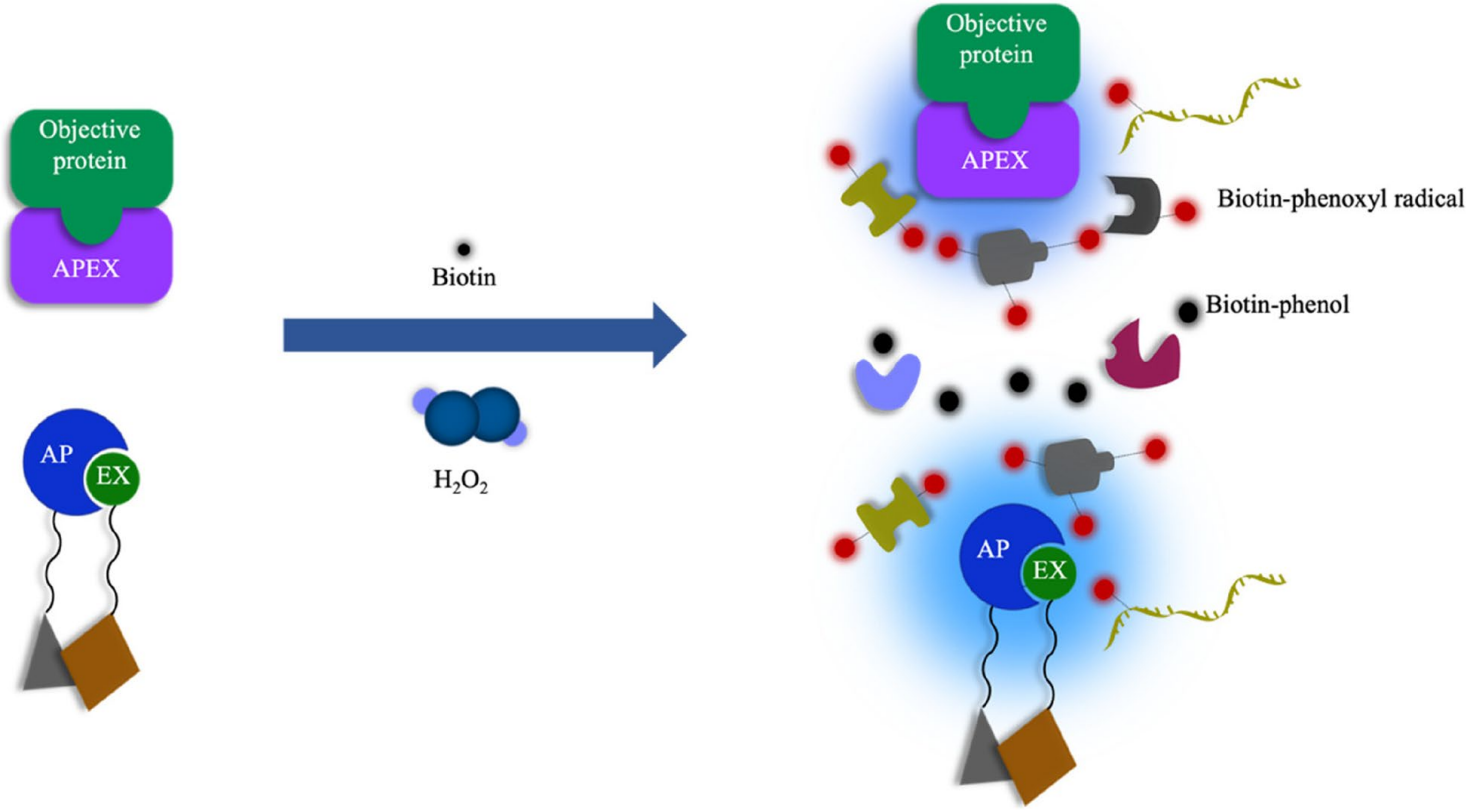
Workflow of APEX and split-APEX. APEX targets organelles or specific protein complexes within cells. After treating living cells with biotin-phenol under H_2_O_2_ conditions for only 1 minute, APEX catalyses the one-electron oxidation of biotin-phenol to form biotin-phenoxy radicals. Split-APEX is divided into two parts, AP and EX, Each fragment has no activity on its own, but when recombined during molecular interactions, peroxidase activity is restored. APEX and split-APEX catalyze the single-electron oxidation of biotin-phenol to form biotin-phenoxy radicals by treating live cells with biotin-phenol for 1 min under hydrogen peroxide conditions

Peroxidases can convert various substrates into radicals in the presence of hydrogen peroxide. Horseradish peroxidase (HRP) was the first proximity labeling enzyme capable of converting aryl azide-biotin reagents into radicals. This method facilitates the study of protein composition in cell membrane domains [34]. Furthermore, when combined with mass spectrometry analysis, it allows the investigation of molecular interactions on the cell surface. In 2012, Martell et al. extracted a new proximity labeling enzyme called APEX from dimeric pea or soybean ascorbate peroxidase [35]. Compared to HRP, APEX lacks disulfide and calcium-binding sites, has a smaller molecular weight (approximately 28 kD), and functions as a monomer. In proteomics, APEX targets organelles or specific protein complexes within cells. The treatment of live cells with biotin-phenol under hydrogen peroxide conditions for just 1 min enables APEX to catalyze the single-electron oxidation of biotin-phenol to form biotin-phenoxyl radicals. This radical can react with water molecules or other radicals, thus limiting the labeling radius to about 20 nm when they diffuse from the peroxidase active site [36]. Moreover, the free radical can react with interacting tyrosine residues within the labeling radius or tyrosine residues of neighboring proteins to be formed into covalent adducts. Although these radicals have an extremely short lifespan, they covalently label endogenous proteins in proximity to APEX. Subsequently, enrichment using streptavidin magnetic beads and mass spectrometry analysis can identify the proteins that interact with the target protein (Fig. 1). Recently, this technique has played a crucial role in determining the protein composition in the human mitochondrial matrix, intermembrane space (IMS) proteome, and mitochondrial calcium uniporter topology.

However, when APEX is expressed at low levels, it becomes challenging to detect diaminobenzidine (DAB) signals by electron microscopy (EM), and biotin-phenol activity is difficult to detect in proteomic analysis. To address this issue, Lam et al. performed directed evolution of APEX, utilizing a yeast display platform and fluorescence-activated cell sorting (FACS) to screen for the most active mutants, resulting in a variant named APEX2 [37]. Studies have shown that although many cells express APEX2 at low levels, it exhibits improved thermal stability and higher biotinylation activity compared to APEX. APEX2 is more active in the reducing cytoplasmic environment and efficiently enriches endogenous mitochondrial and endoplasmic reticulum proteins while tolerating high concentrations of H_2_O_2_, among other advantages [38]. APEX2 is also more sensitive when applied in electron microscopy because its contrast generation does not require light, making it easier to use as an EM reporter than other tags, such as mini singlet oxygen generator (miniSOG), resorufin-based label (ReAsH) and fluorescent proteins [39, 40]. With APEX2, staining can be achieved across large fields of view without the need for special equipment. This method can replace indirect measurement techniques (such as subcellular fractionation) and can be used for protease accessibility tests or protein blotting to precisely determine the subcellular localization and membrane topology of important proteins [41].

**Fig. 2:**
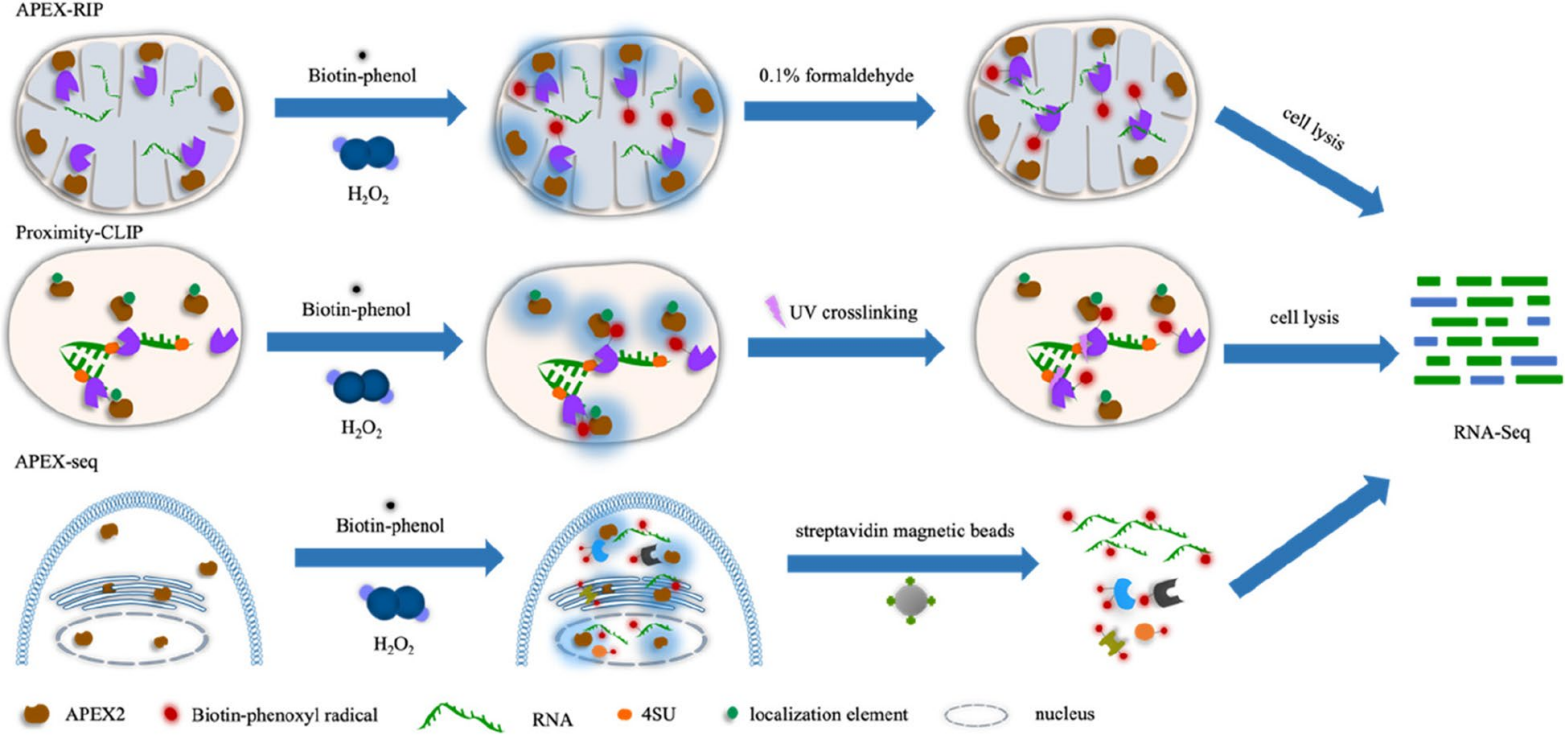
Workflow of APEX-RIP, Proximity-CLIP and APEX-seq. APEX-RIP: Cells expressing APEX2 were treated with biotin-phenol and hydrogen peroxide to biotinylate proximal endogenous proteins. The biotinylated proteins were then cross-linked to nearby RNA using 0.1% formaldehyde. After cell lysis, the biotinylated nucleic acids were enriched by streptavidin and finally analyzed by RNA-Seq. Proximity-CLIP: RNA was first labelled with 4SU and APEX2 was fused to the localisation element (targeting to the compartment of interest), followed by treatment of cells with biotin-phenol and hydrogen peroxide to biotinylate the proximal protein. Protein-RNA cross-linking was then achieved using UV light (λ > 312 nm). Purified protein-RNA complexes after cell lysis can be used for protein mass spectrometry and RNA-Seq analysis. APEX-seq: APXE2 was first localised in the cytoplasm, cytoplasmic face of the endoplasmic reticulum membrane or nucleus, followed by initiation of APEX2 to biotinylate the proximate RNA. After affinity purification of the above RNA by streptavidin peroxidase, RNA sequencing analysis proceeded

Currently, APEX and APEX2 are used not only in proteomics but also for studying transcriptomes of subcellular compartments. In 2017, Kaewsapsak et al. combined the catalysis of spatially restricted in situ protein biotinylation by peroxidase with RNA‒protein chemical crosslinking, resulting in a method named APEX-RIP, which allows the highly specific and sensitive isolation of RNA from various subcellular compartments, such as the mitochondrial matrix, cell nucleus, cytoplasm, and endoplasmic reticulum [42]. In 2018, Benhalevy et al. developed Proximity-CLIP using APEX2-mediated specific biotinylation of proteins and ultraviolet (UV) crosslinking. This method enables the study of RNA and proteins in the cell nucleus, cytoplasm, and cell‒cell interfaces. Proximity-CLIP offers significant advantages in RNA binding protein (RBP)-protected footprint sequencing, as it allows not only the analysis of localized RNA but also the identification of cis-acting elements occupied by proteins on RNA [43] 0.2019 Alejandro et al. developed APEX-seq, a proximity labeling technique for exploring RNA, using APEX2. Because nucleotides are also amenable to free radical reaction chemistry, RNA can be biotinylated with APEX2. The method involves targeting the APEX2 peroxidase gene to the relevant region in living cells. Biotin-phenol (BP) was then added under H_2_O_2_ conditions for 1 min, leading to biotinylation of endogenous proteins and RNAs within a few nanometres of APEX2. The biotinylated RNA was then isolated using streptavidin magnetic beads and analyzed by poly(A) selection and RNA sequencing (RNA-seq) [44]. Fazal et al. conducted further research using APEX-seq to investigate the spatial localization and complete sequences of thousands of endogenous RNAs in live cells, revealing the extensive localization patterns of different RNA categories and transcript isoforms [45]. However, this technique has two drawbacks, one is that it requires recombinant expression of the APEX-fusion construct in the target cells, and the other is that it is difficult to label RNAs within macromolecular complexes. The workflows of APEX-RIP, Proximity-CLIP and APEX-seq are illustrated in Fig. 2.

To address the issue of off-target labeling in proximity labeling techniques, Han et al. developed a split-APEX (sAPEX) approach in 2019 [46]. The enzyme is divided into two parts, AP and EX, with AP being a 200-amino acid N-terminal fragment selected from a yeast display library and EX being a 50-amino acid C-terminal fragment. Each fragment has no activity on its own, but when recombined during molecular interactions, peroxidase activity is restored (Fig. 1) [47]. Split-APEX technology has been applied to mammalian cell membranes, noncoding RNA scaffolds, and mitochondria-associated endoplasmic reticulum contact sites [46]. Currently, APEX proximity labeling technology is an ideal method for precisely monitoring local protein environment changes. For example, it can be used to explore cell responses to changes in drug concentrations over time, growth conditions, or temperatures with rapid labeling kinetics (labeling time required is less than 1 min) [48]. However, this method requires the use of low-membrane-permeable biotin-phenol for oxidative stress and toxic H_2_O_2_ to terminate the reaction, making it challenging to apply in living organisms. In 2020, Han et al. proposed an alternative method for mapping RNA‒protein interactions inside live cells by combining the MS2-MCP system or CRISPR-Cas13 system [49] with the APEX2 proximity labeling enzyme, targeting specific RNAs. This approach allows the high-specificity delivery of APEX2 to human telomerase RNA (hTR), enabling the study of interactions between hTR and its associated proteins [50]. However, this method has only been demonstrated on overexpressed, highly abundant cellular RNAs.

## Proximity labeling techniques based on biotin Ligases

### BioID, BioID2 and Split-BioID

**Fig. 3:**
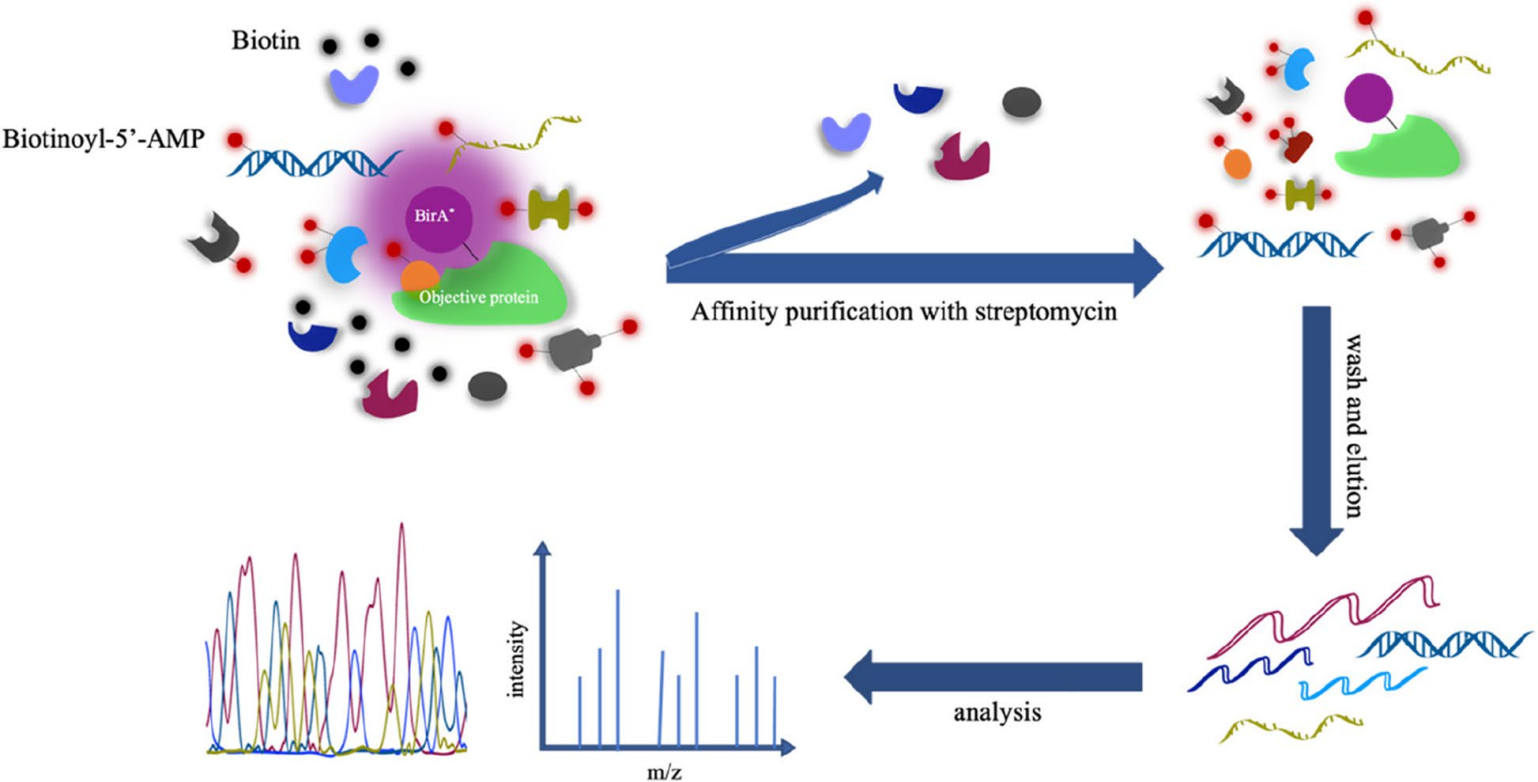
Workflow of BioID and TurboID. BioID is a humanized version of the BirA protein from E. coli with a R118G mutation, TurboID is a directed-evolution variant of BioID. Here we mark them as BirA*. When BirA* is fused to a target protein and expressed in cells, exogenously added free biotin can be converted to highly reactive biotin-5'-AMP. Within the labeling radius, proteins that either directly or indirectly interact with the fusion protein will be labeled. Subsequent selective isolation and identification of these biotinylated proximal endogenous proteins using biotin affinity purification. Finally, the resulting proteins are identified and analyzed by mass spectrometry

In 2004, Choi-Rhee et al. mutated the 118th arginine (Arg) residue of the Escherichia coli biotin protein ligase BirA to glycine (Gly), resulting in R118G [51]. This mutation not only reduced BirA’s affinity for Bio-5’-AMP but also allowed biotinylation of surrounding molecules without requiring specific amino acid sequence recognition. In 2012, Roux et al. utilized the aforementioned mutated BirA to develop the first proximity labeling technique using the biotin ligase, called BioID, with a molecular weight of 35 kDa [52]. This mutation allows BirA to covalently label specific lysine residues on the surface of target proteins with biotin-5’-AMP, selectively biotinylated acetyl-CoA carboxylase. When the BioID ligase is fused to the target protein and expressed in cells, it converts exogenously added free biotin into a highly reactive but unstable biotin-5’-AMP, which is released from the enzyme’s active site and reacts with nearby protein amines. Proteins within a labeling radius of 10 nm, whether directly or indirectly interacting with the fusion protein (through additional protein‒protein interactions), will be labeled, while distal proteins, regardless of their interaction with BioID, will remain unlabeled [53]. Subsequently, biotin affinity purification can be used to selectively isolate and identify these biotinylated proximal endogenous proteins. Finally, the proteins are obtained by mass spectrometry identification and analysis (Fig. 3). The R118G mutation reduces the affinity of BioID for biotin and bioAMP (approximately 40-fold and 440-fold lower than that of the wild type, respectively), leading to a significant increase in the release of active bioAMP molecules. This enables BioID to covalently label lysine residues on proteins within a 10 nm radius. [54]. Additionally, in the absence of sufficient biotin (5–50 µM), the reduced biotin affinity of BioID may decrease biotinylation. The initiation of biotinylation can be temporally controlled to achieve controlled mixed labeling for selective or comparative studies [55]. As BioID identifies protein interactors of the target protein, it can be used for the identification of insoluble proteins and the study of weak and transient protein‒protein interactions [56–58].

In view of the large molecular weight of BioID, which hinders its targeting in certain fusion proteins, in 2016, Kim et al. developed a smaller enzyme called BioID2 with a molecular weight of 27 kDa, derived from *Aquifex aeolicus* [16]. Compared to BioID, BioID2 offers the following advantages: it targets fusion proteins with greater selectivity, requires less supplementary biotin, and exhibits enhanced labeling of neighboring proteins. BioID2 not only improves the screening efficiency of protein‒protein interactions but also allows specific modulation of the biotin labeling radius [59]. In 2017, Schopp et al. split BirA into two protein fragments to create Split-BioID, with a molecular weight of 35 kDa. This method is part of conditional proteomics, where a quantifiable active protein is divided into two poorly interacting nonfunctional fragments [60]. When fused to two interacting proteins, the fragments can reassemble to regain activity. Split-BioID enables the validation of protein‒protein interactions and simultaneously labels other neighboring proteins belonging to the respective complexes in live cells, thereby compensating for the limitations of affinity purification and BioID methods [61].

### TurboID, miniTurbo, Split-TurboID and ProtA-TurboID

BioID, BioID2, and Split-BioID have relatively long labeling times (16–18 h), which may affect the labeling of transiently interacting proteins and protein functions, leading to false-positive or false-negative results. Additionally, their efficiency is lower at temperatures below 37 degrees Celsius. In 2018, Branon et al. used yeast display, directed evolution, Tyramide Signal Amplification (TSA), linker enzyme reduction removal, and negative selection to generate two new linker enzymes: TurboID and miniTurbo. TurboID has a size of 35 kDa [62]. Both TurboID and miniTurbo biotinylate proteins at a faster rate without compromising specificity. The biotinylation products labeled by TurboID or miniTurbo within 10 min are comparable to those labeled by BioID/BioID2 within 18 h [62, 63]. Moreover, they can function effectively at lower temperatures. TurboID has 15 mutated base pairs compared to wild-type BirA, allowing it to use ATP to convert biotin to biotin-AMP, a reactive intermediate that subsequently covalently labels nearby proteins (Fig. 3). Due to evolution through the yeast secretory pathway, TurboID exhibits significantly higher activity in the endoplasmic reticulum lumen than BioID [56].

Studies have shown that TurboID is the most active biotin ligase, and it can utilize endogenous biotin in certain cells and organisms, displaying biotinylation activity even before exogenous biotin addition [62]. On the other hand, miniTurbo, with a molecular weight of only 28 kDa, has an N-terminal structural domain deletion and 13 bp mutations compared to wild-type BirA. This further reduces the potential interference with fusion protein transport and function. Although miniTurbo’s activity is 1.5-2 times lower than that of TurboID, it only labels a small amount of proteins before adding exogenous biotin, indicating that miniTurbo allows control over the labeling window time [56]. Furthermore, TurboID and miniTurbo perform better in vivo than BioID because BioID originates from *Escherichia coli* (grown at 37 degrees Celsius), while TurboID and miniTurbo evolved in yeast (grown at 30 degrees Celsius).

**Fig. 4:**
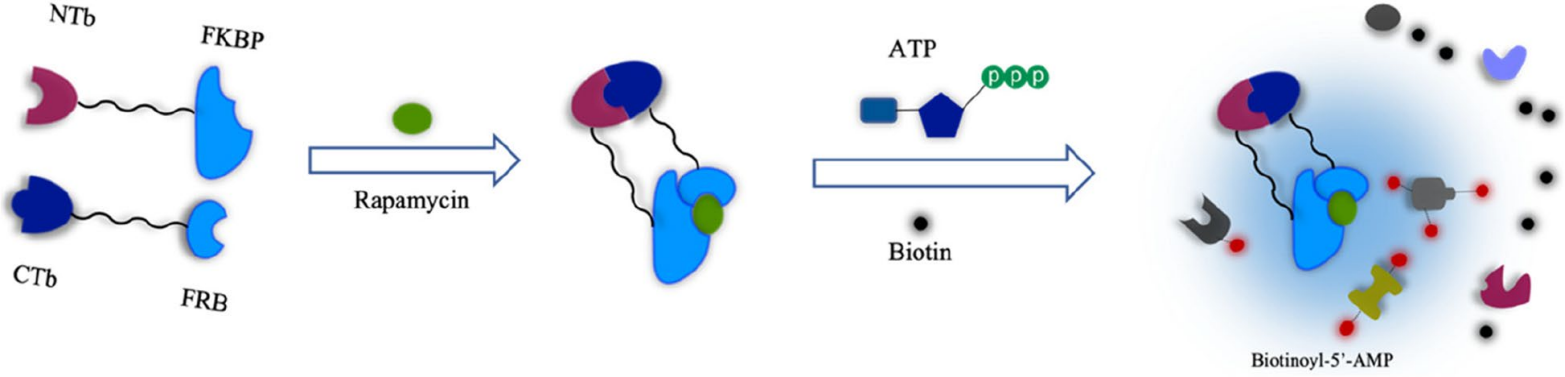
Workflow of Split-TurboID. Split-TurboID was applied to the FRB-FKBP dimer system, and after treatment with rapamycin, the two inactive fragments of TurboID recombined to form an active enzyme that produced biotin-5’-AMP

Traditional proximity labeling methods have limitations in targeting specificity (cannot obtain specific protein complexes or locate organelle contact sites) and may not tolerate high-molecular-weight protein fusions. To address these issues, researchers have split the labeling enzymes. Split-APEX and split-BioID were introduced earlier, but split-APEX requires the addition of H_2_O_2_ and ferrous heme, limiting its in vivo use, while split-BioID has very low activity. To solve the above problems, in 2020, Cho et al. combined two nonactive fragments of TurboID and named it split-TurboID, which has a molecular weight of 35 kDa [64]. These two nonactive fragments can reassemble through protein‒protein interactions or organelle-organelle interactions. The authors screened 14 different TurboID split points to identify the best fragments for high- and low-affinity recombination. Eventually, they chose TurboID split at L73/G74, which gave rapamycin-dependent reconstitution when fused to FKBP12-rapamycin-binding (FRB) and FK506-binding protein (FKBP) in multiple subcellular organelles. Upon rapamycin treatment, split-TurboID recombines to form an active enzyme that produces biotin-5′-AMP for proximity-dependent labeling. The N-terminal fragment NTb was fused to FKBP and V5. The C-terminal fragment CTb was fused to HA, HaloTag and FRB (Fig. 4). Split-TurboID comes in two forms: low affinity and high affinity [65, 66]. Upon biotin incubation for less than 1 h, both methods can catalyze proximity labeling, and their activity is not only much higher than that of split-BioID but also higher than that of full-length BioID.

In 2021, Santos-Barriopedro et al. developed a proximity biotinylation method called ProtA-TurboID, which does not require additional mutations [67]. This enzyme is a recombinant fusion of the biotin ligase TurboID and the antibody recognition molecule ProteinA. The principle of this method involves adding bait-specific antibodies and ProteinA-Turbo enzyme consecutively to permeabilized or nonpermeabilized cells. After incubation, Protein A-Turbo antibody-antigen complexes form, and unbound molecules are washed away. Subsequently, exogenous biotin is added to trigger bait-proximal biotinylation. Finally, streptavidin magnetic beads are used to enrich biotinylated proteins from crude lysates, followed by mass spectrometry-based protein identification. ProtA-TurboID can, in principle, determine the proximal proteome of any target protein within 3 days. Since it does not require transfection, transduction, or other genetic manipulations of the target cells, ProtA-TurboID can potentially be applied to any cell type. Additionally, if specific antibodies recognizing posttranslational modifications can be obtained, the ProtA-Turbo enzyme can also be used for proximity proteomics studies.

### BASU

BASU is a new mutant with a molecular weight of 29 kDa designed from *Bacillus subtilis*. Compared to the previously standard *Escherichia coli* BirA, its kinetic rate was increased by over 1000-fold, and the signal-to-noise ratio was improved by over 30-fold. It enables the direct study of RNA‒protein interactions in live cells within 1 min [68]. In 2020, Villaseñor et al. used BASU to establish a ChromID method, which fuses BASU to engineered chromatin readers (eCRs). This method is used to identify proteins interacting with individual histone marks, such as trimethylated histone 3 lysine 9 (H3K9me3), trimethylation of lysine 4 on histone H3 (H3K4me3), and trimethylation at lysine 27 of histone H3 (H3K27me3) [69].

### AirID

In 2020, Kido et al. reconstructed the BirA algorithm and constructed a new proximity-dependent biotin labeling enzyme called AirID using metagenomic data. Fusion proteins such as AirID-p53 or AirID-IB were used to biotinylated mouse double minute 2 (MDM2) or RelA in vitro and in cells, respectively. AirID-CRBN demonstrated biotinylation of IKAROS Family Zinc Finger 1 (IKZF1) and spalt-like transcription factor 4 (SALL4) in a pomalidomide-dependent manner. Through streptavidin pull-down experiments, AirID-CRBN was able to biotinylate endogenous cullin 4 (CUL4) and RING-box protein 1 (RBX1) in the CUL4-DDB1-CRBN-RBX1 (CRL4CRBN) complex. Stable expression of AirID-IB in cells and subsequent liquid chromatography-tandem mass spectrometry/mass spectrometry (LC‒MS/MS) analysis revealed high-level biotinylation of the RelA protein, indicating that AirID is a novel enzyme for protein‒protein interaction analysis. Although its sequence similarity with BioID is 82%, AirID exhibits higher biotinylation activity toward interacting proteins in both in vitro and cellular experiments [21].

## Other proximity marking techniques

### PUP-IT

Pupylation-based interaction tagging (PUP-IT) is a novel method developed by Liu et al. in 2018 to study membrane protein interactions [70]. This method utilizes the bacterial Pup protein conjugation system, where the PafA gene encoding the Pup ligase is fused to the bait protein [71]. This allows the enrichment and mass spectrometry detection of transient and weakly interacting proteins. Pup is a small bacterial protein consisting of 64 amino acids with a Gly-Gly-Gln motif at the C-terminus. Additionally, activated Pup proteins cannot freely diffuse from the PafA ligase, ensuring high specificity of the labeling. However, PUP-IT may not be suitable for interactions within organelles, as the relatively large size of Pup prevents transmembrane diffusion [72].

## Applications of the proximity labeling technique

### Applications of peroxidase-based proximity labeling technology

Various ribonucleoprotein complexes control mRNA processing, translation, and decay. These complexes localize transcripts to specific regions within the cell and can condense into non-membrane-bound structures. Studying these structures is crucial for understanding cell function and intercellular signaling. Zhou et al. applied biotin-phenylamine (Btn-An)-based APEX2 labeling to the mitochondrial matrix and found that all 13 mitochondrial messenger RNAs were labeled, while no cytoplasmic RNA was labeled. Moreover, APEX2-mediated Btn-An labeling showed high spatial specificity in other subcellular compartments, such as nucleoli and nucleoplasm. Therefore, APEX2 probes can be used for DNA/RNA labeling. APEX2 can also crosslink RNA to biotinylated proteins (APEX-RIP and Proximity-CLIP) to analyze subcellular transcriptomes [42, 43]. Currently, these techniques have been successfully applied to label RNA in various subcellular compartments, including the mitochondrial matrix, cell nucleus, and endoplasmic reticulum. In principle, APEX-RIP preferentially labels RNA with more bound proteins, while APEX2-mediated RNA biotin-phenylamine labeling is advantageous for preferentially capturing RNA with fewer bound proteins. However, APEX-RIP and Proximity-CLIP cannot directly target specific bases, and additional crosslinking steps are necessary after APEX2 labeling, decreasing the spatial resolution [73, 74].

In mammalian cells, the nucleus contains copper, and abnormal copper accumulation occurs during cell carcinogenesis. However, the mechanisms of nuclear copper accumulation and the broader functional importance of copper remain unclear. Therefore, in 2021, Chen et al. used APEX2 technology to explore the neighboring proteins of the copper chaperone Atox1 and conducted mass spectrometry analysis to identify a new nuclear copper-binding protein called cysteine-rich protein 2 (CRIP2), which interacts with Atox1 in the cell nucleus. Upon copper transfer from Atox1 to CRIP2, CRIP2 undergoes secondary structural changes, ultimately promoting its ubiquitin-mediated protein degradation. Furthermore, CRIP2 depletion and copper-induced CRIP2 degradation increase reactive oxygen species (ROS) levels and activate autophagy in H1299 cells. Therefore, this study identified CRIP2 as an autophagy-inhibiting protein and linked CRIP2-mediated copper metabolism to autophagy in cancer cells [75].

Due to the specificity and sensitivity of APEX-seq for mitochondrial matrix and ezrin-radixin-moesin (ERM) proteins, Fazal et al. applied this method to seven compartments, namely, the nucleolus, nuclear pores, nuclear lamina, endoplasmic reticulum (ER) lumen, ER membrane, mitochondrial matrix, and outer mitochondrial membrane (OMM), to construct RNA maps. Since these regions are difficult to purify or too small for conventional microscopy imaging, determining the RNA content in these compartments presents substantial challenges. APEX-seq allows the connection of previously isolated RNAs to these compartments, enabling observation of RNA localization at different cellular positions [44]. Their RNA maps revealed that 324 RNAs localized to the nucleolus, 114 to the nuclear lamina, and 111 to the OMM [45]. When Alejandro et al. employed APEX-seq, they found that it not only resolved the localization of intracellular RNAs but also determined their enrichment or depletion near key RNA-binding proteins. Furthermore, matching the spatial transcriptome revealed by APEX-seq with the spatial proteome determined by APEX mass spectrometry (APEX-MS) provided new insights into the organization of active mRNA translation initiation complexes and the composition of stress granules [76].

## Applications of biotin ligase-based proximity marker technology

Biotin ligase-dependent proximity labeling techniques have been widely applied in protein‒protein interaction studies and in determining the proteomic localization of cellular structures, including nuclear pore complexes, transcription regulatory complexes, Hippo pathway components, stress granules, cilia, centrosomes, pathological protein aggregates, and interactions with viruses and pathogens [33, 66, 77–81]. These proximity labeling methods have also been used in various cell types and species, including bacteria, yeast, plants, flies, worms, and mice [82–86].

### Applications of BioID, BioID2 and Split-BioID

Due to its high spatial resolution, BioID is suitable for screening protein‒protein interactions in live cells [87], especially for insoluble, weak, and transient interactions. Currently, it is widely used in the study of cell‒cell connections, spatial dynamics of epigenetic factors, cancer development, mRNA decay, signaling pathway regulation, and ubiquitin metabolism in various cellular mechanisms [88–92].

#### Applications in mammals

BioID methods have great potential in mammalian subcellular and cell-specific proteomics. While the purification and analysis of excitatory postsynaptic protein complexes have provided a foundation for neurobiology research, little is known about the inhibitory postsynaptic density (iPSD). In 2016, Uezu et al. developed adeno-associated viral (AAV) constructs fused with Gephyrin and PSD-95 to label proteins associated with inhibitory and excitatory postsynaptic membranes, respectively, in tissues in vivo. This approach revealed 140 previously unidentified proteins interacting with iPSD, including a wide range of signaling, transmembrane, structural, and uncharacterized proteins [93]. Proximity labeling techniques can also be used to monitor miRNA. In 2017, Schopp et al. used Split-BioID to investigate the miRNA-mediated silencing pathway and to identify the protein interactomes of Argonaute 2 (Ago2) in two different functional complexes. They discovered that Grb10-interacting GYF protein 2 (GIGYF2) is a regulator of miRNA-mediated translation repression [61].

Phosphorylation of the Bcl2 family protein Bid can increase apoptosis initiation and sensitivity to mitotic drugs. To explore the dynamic coordination of the Bcl2 protein in apoptosis initiation, in 2020, Pedley et al. constructed an mBid-BirA fusion protein. BioID catalyzes the in situ biotinylation of proteins, allowing the extraction of intact membrane components and preventing detergent-induced abnormal interactions of Bcl2 proteins. Phosphorylated full-length BH3-interacting domain death agonist (FL-Bid) reversibly increases apoptosis initiators during mitosis, and Bid phosphorylation during mitosis-induced apoptosis initiation depends on voltage-dependent anion-selective channel protein 2 (VDAC2), suggesting that the Bcl2 family interaction network plays a crucial role in regulating the timing of apoptosis initiation [32].

Secreted factors that directly or indirectly act between organs are key regulatory factors in encoding system homeostasis. However, the ability of traditional methods to identify regulators of endogenous secreted factors is limited. In 2021, Droujinine et al. designed and applied highly active BirA to fruit flies and discovered 51 muscle-secreted proteins in the head and 269 fat-secreted proteins in the legs/muscles, including CG2145, a human homolog endoribonuclease (EndoU), which directly binds to muscles and promotes muscle activity [94]. In the same year, Pronobis et al. used BioID2 to study changes in the proteome of zebrafish cardiomyocytes after large-area cardiac injury. They not only identified Rho A as a target of the human epidermal growth factor receptor 2 gene (ErbB2) signaling pathway in cardiomyocytes but also found that blocking Rho A during heart regeneration (or mitotically induced heart stimulations) resulted in changes in neuregulin 1 (Nrg1), Vegfaa, or vitamin D, disrupting muscle regeneration. This study demonstrated that proximity labeling techniques can be used for the identification of cellular proteomes and signaling pathway networks [95].

In 2021, Kannangara et al. investigated the only multispanning transmembrane protein, autophagy-related protein 9 A (ATG9A), in the core autophagy-related proteins (ATGs). They fused BioID to the C-terminus of ATG9A and established stable cell lines expressing HA-tagged BioID and ATG9A-BioID. Subsequently, they used streptavidin affinity resin to capture biotinylated proteins and performed initial evaluations using Coomassie staining on the captured proteins. Finally, they revealed the network of interactions involving ATG9A through quantitative mass spectrometry analysis. The authors found that this network not only included members of the UNC-52-like kinase 1 (ULK1) complex, membrane fusion, and vesicle transport regulatory factors but also involved TRAnsport Protein Particle (TRAPP), endosome-associated recycling protein (EARP), glycoprotein A repetitions predominant (GARP), exocyst, AP-1, and AP-4 complexes. These data revealed the role of the ULK1-independent autophagy-related protein 13 (ATG13) complex in regulating ATG9A and the interactions of ATG9A in vesicle transport and autophagy pathways [19].

#### Application in plants

BioID has become a powerful tool for studying mammalian proteomes, including insoluble and membrane-associated proteins. Recent research has shown that it can also efficiently screen for interacting and neighboring proteins in plant cells [86]. However, compared to mammalian systems, the application of this technology in plants is not yet widespread. This might be due to specific structural features in plants that can interfere with protein detection and identification [96], such as the relatively small cytoplasmic volume compared to the cell wall mass and the high levels of proteases and phosphatases.

In 2017, Lin et al. first reported the establishment of the rice protoplast BioID system. The authors disrupted a cryptic internal split site in BirA and named the resulting protein BirAG. They subsequently used this method to study the neighboring or interacting proteins of the rice transcription factor OsFD2. By employing three background-reducing control groups, the authors determined that OsFD2 is proximal to 62, 30, and 12 proteins, respectively. They also found that the degree of biotinylation of proteins in rice protoplasts increased with the expression level of BirAG, and the biotinylation efficiency was enhanced with increasing culture time and the addition of high concentrations of exogenous biotin [97].

In 2018, Khan et al. applied BioID to the model plant *Arabidopsis thaliana*. They focused on HopF2bPt0DC3000 (HopF2), a membrane-targeted type III secretion effector that promotes the growth of *Pseudomonas syringae* in Arabidopsis. Under the control of a dexamethasone-inducible promoter, the authors generated transgenic Arabidopsis plants that could specifically express HA epitope-tagged HopF2-BirA and BirA. By subsequently adding biotin (directly infiltrating biotin into leaf tissues) and performing streptavidin affinity purification, LC‒MS/MS analysis, and other methods, the authors identified over 500 proteins from each sample. Next, using Significance Analysis of INTeractome (SAINT) analysis, they found that 39 proteins were labeled by HopF2-BirA, and 111 proteins were labeled by BirA. Among these proteins, 19 were specific to HopF2-BirA labeling, and 91 were specific to BirA labeling. This research opens up new avenues for studying the plant proteome [85].

In 2018, Conlan et al. investigated the interactions of the effector protein AvrPto from the tomato pathogen *Pseudomonas syringae pv.* tomato (Pst) with various plant immune-related proteins. To explore the interactions between AvrPto and neighboring immune system proteins, the authors designed four constructs: AvrPto_BirA (fusion of AvrPto with the N-terminus of BirA), MP_BirA (fusion of an 8-amino acid AvrPto myristoylation/palmitoylation (MP) motif with the N-terminus of BirA), BirA alone, and an empty vector (EV) control. This design not only allowed arbitrary protein interactions on the plasma membrane to be distinguished but also identified target-specific protein interactions. The authors used mass spectrometry to analyze the four constructs and identified 271 proteins in total. Among them, 61 proteins were identified in the EV negative control sample, 150 proteins in the BirA control group, and 60 proteins in both the AvrPto_BirA and MP_BirA samples, with limited overlap between the latter two. The authors identified five AvrPto-proximal plant proteins from these interactions and studied their impact on plant immune function and growth [98].

In 2019, W. Macharia et al. investigated the relationship between RNA viruses and plant autophagy by fusing BirA to AuTophaGy-related 8 (ATG8) and conducting mass spectrometry analysis. They compared the autophagy induction rate in *Nicotiana benthamiana* plants infected with *Tobacco mosaic virus* (TMV) and its mutant TMV24A + upstream pseudoknot domain (TMV24A + UPD), which induces earlier and more severe necrotic symptoms. The results showed that compared to TMV, TMV24A + UPD induced an increased autophagy flux. Under TMV24A + UPD infection, the authors identified a large number of ATG8-interacting proteins, among which NbHYPK was a newly discovered ATG8-interacting molecule. This provides new insights for researchers regarding the effectiveness of autophagy in a compatible virus‒host interaction [99].

#### Applications in microbiology

BioID provides a novel approach to exploring protein proximity and interactions. It has been successfully applied in model organisms such as fruit flies, zebrafish, mice, peas, other mammals, and plants. As research demands grow, this technology is increasingly used in microorganisms as well. *Trypanosoma brucei*, a highly characteristic parasitic protozoan, plays a significant role in biology due to its complex and highly organized cytoskeleton. However, traditional methods have limited the study of protein components in the *T. brucei* cytoskeleton. In 2013, Morriswood et al. used BioID to investigate novel bilobe components in *Trypanosoma brucei*. They tagged the bilobe marker protein *Trypanosoma brucei* MORN1 (TbMORN1) with Myc-BirA and then identified a large number of proteins interacting with TbMORN1 through techniques such as biotinylated protein purification and mass spectrometry. This study revealed new structural components of the cytoskeleton, providing important insights into its organization of discrete subdomains [100].

The inner membrane complex (IMC) of *Toxoplasma gondii* is a unique organelle composed of two distinct elements: flattened membrane sacs called alveoli located beneath the parasite plasma membrane and a rigid cytoskeletal network supported by intermediate filaments. The IMC plays a crucial role in parasite motility, host cell invasion, and intracellular replication. In 2015, Chen et al. utilized BioID technology to identify proteins in Toxoplasma and used the IMC membrane protein ISP3 as bait to identify new IMC proteins. This approach not only provided a new method for identifying novel proteins in subcellular compartments of Toxoplasma but also laid a solid foundation for assessing protein interactions within the IMC [101].

In 2020, Bradley et al. also employed BioID to study Toxoplasma. The authors fused BioID (or BioID2) to a bait protein using endogenous gene tagging in Toxoplasma and then identified interacting and neighboring proteins within subcellular compartments through in vivo biotinylation, streptavidin affinity purification, and mass spectrometry [83].

Protein‒protein interactions between viral proteins and host proteins play a crucial role in viral invasion, replication, and inhibition of host immune responses. However, traditional protein interaction techniques have limitations and may not provide comprehensive proteomic information. Herpesviruses are important human pathogens that cause a wide range of diseases, from skin lesions to malignant tumors. Therefore, defining and characterizing the composition of protein complexes is essential for understanding the virus replication mechanism and discovering potential therapeutic targets. In 2020, Cheerathodi et al. utilized BioID in combination with mass spectrometry to study the protein‒protein interaction network of herpesvirus [82]. In 2021, Chen et al. used BioID2 to study the function of host‒virus protein‒protein interactions in severe acute respiratory syndrome coronavirus 2 (SARS-CoV-2), which identified 437 interacting proteins with high confidence. Studying these interactions laid a solid foundation for elucidating the involvement of SARS-CoV-2 viral proteins in host cell life cycle processes [102]. In 2022, May et al. generated stable A549 human lung cancer cells expressing BioID-tagged SARS-CoV-2 viral proteins to explore the global proteomic changes induced by viral protein expression and the specific protein‒protein interactions between individual SARS-CoV-2 viral proteins and host cell proteins. The authors not only confirmed previous findings, such as the role of open-reading frame 3a (ORF3a) in extensive membrane remodeling and viral budding through interactions with VPS39 and VPS11, but also identified potential interactions between ORF3a and cell adhesion factors. Moreover, by cross-referencing the BioID dataset with clinical and FDA-approved drug libraries, the authors discovered potential drugs related to coronavirus therapy [88].

### TurboID, miniTurbo and Split-TurboID

#### Applications in mammals

The recent literature indicates significant developments in the application of TurboID and miniTurboID in animal studies. This is because while BioID originates from *Escherichia coli* (37 °C), TurboID and miniTurboID are derived from yeast (30 °C), making them more suitable for use in model organisms such as flies and worms [62, 103–105]. In studies focusing on flies, researchers compared the expression efficiency of different biotin ligases in specific regions of larval wing discs [106]. The results showed that TurboID and miniTurbo exhibited higher catalytic activity than BioID, with 22-fold and 10-fold increases, respectively [62, 106]. This improvement significantly enhances the stability and reliability of the technique. Additionally, all three biotin ligases were tested in worm experiments, where TurboID and miniTurbo demonstrated better activity than BioID. In worm experiments, TurboID showed higher expression levels than miniTurbo in adult worms, resulting in stronger labeling effects. Furthermore, increasing the temperature can further enhance the biotinylation efficiency of TurboID and miniTurbo [62]. However, in some cases, the high activity and biotin affinity of TurboID may lead to cell toxicity. For instance, constitutive expression of TurboID in fly tissues can cause excessive biotin depletion, leading to decreased survival rates and smaller body size without additional biotin supplementation. This toxicity can be improved by supplementing biotin, inducing TurboID expression, or limiting TurboID expression to specific tissues or organs [107]. Moreover, prolonged TurboID labeling for more than 24 h in cultured mammalian cells (normal TurboID allows covalent labeling within 10 min) can lead to overbiotinylation of the endogenous proteome and result in growth defects [56]. Therefore, low-activity proximity labeling methods, such as BioID or AirID, may be more advantageous in long-term experiments or experiments with biotin supplementation challenges.

In addition to the aforementioned studies, TurboID, miniTurbo, and Split-TurboID play crucial roles in protein complex and cellular structure proteomic localization. They have been used in various aspects, such as signal transduction pathways, protein‒protein interaction network identification, DNA-binding protein exploration, and synaptic function exploration [108–112]. Studies have shown that Lck plays a key role in the initiation of the T-cell receptor (TCR) signaling pathway. However, the precise regulation of Lck in T cells after TCR activation remains to be investigated. Yu et al. expressed Lck-TurboID in Jurkat T cells and obtained a dynamic, short-range Lck protein interaction network after 30 min of TCR stimulation. They detected 27 early signal-induced Lck proximal interactors in live T cells. This study revealed previously overlooked Lck PPIs, which may be associated with cytoskeletal rearrangement, the ubiquitination of TCR signaling proteins, the activation of mitogen-activated protein kinases, the coalescence of LAT signalosomes, and the formation of the immune synapse [113].

In 2021, Barroso-Gomila et al. fused one fragment of Split-TurboID with Small Ubiquitin-like Modifier (SUMO) and another fragment with the protein of interest. In the presence of biotin, the TurboID recombinant becomes specific, enabling it to biotinylate interacting partners in a SUMO-dependent manner. The transient SUMO-dependent interacting complex was then identified through streptavidin affinity purification and LC‒MS analysis. With this method, the authors revealed the role of SUMOylation in promyelocytic leukemia nuclear bodies (PML NBs) in early promyelocytic leukemia (PML) and discovered 59 SUMO-dependent PML-interacting proteins. These proteins are involved in essential nuclear processes such as protein SUMOylation, transcriptional regulation, and DNA repair [114].

In 2021, Santos-Barriopedro et al. utilized ProtA-TurboID to discover the interaction between the FLYWCH/Zn-finger DNA-binding domain (FLYWCH1) and a subset of centromeres marked by H3K9Me3 in human cells [67]. Currently, the ProtA-Turbo enzyme is mainly used for protein studies in the cell nucleus and nuclear membrane. However, by employing additional steps such as blocking endogenous biotinylated proteins with free streptavidin, subcellular fractionation, or extra washes, this method may potentially be applied to study proteins in the cytoplasm or plasma membrane [115].

In 2023, Fei et al. developed a proximity labeling method based on the TurboID enzyme to study proteins interacting with specific DNA sequences. The authors first placed tetracycline response element (TRE) sequences next to the target DNA sequence, as TRE sequences have high affinity for the tetracycline-controlled transcriptional activator (tTA) protein. Next, they fused the binding partners FKBP and FRB, which are regulated by rapamycin, with TurboID and tTA proteins, respectively. In the presence of rapamycin, the TRE sequences attracted TurboID, which biotinylated nearby proteins. The biotinylated proteins were then detected using LC‒MS [116]. Perisynaptic astrocytic processes are essential components of central nervous system synapses. However, the molecular mechanisms controlling astrocyte–synapse adhesion and how astrocyte contacts regulate synaptic formation remain incompletely understood. In 2020, Takano et al. used Split-TurboID to identify the proteome enriched at astrocyte–neuron junctions in vivo, including neuronal cell adhesion molecule (NrCAM). They found that NrCAM is expressed in cortical astrocytes and is necessary to restrict neuronal infiltration through astrocytic processes. This study provided a proteomic framework for the astrocyte–neuron interface and revealed the role of astrocytes in controlling the formation of GABAergic synapses [66].

Currently, the composition of many cell proteomes in vertebrates remains unknown. Zebrafish is a commonly used model organism for vertebrate research. In 2021, Rosenthal et al. applied proximity labeling techniques to zebrafish and designed the bait protein lamin A (LMNA) and the negative control GFP (green fluorescent protein) to establish TurboID and miniTurbo labeling in early zebrafish embryos and optimized and compared them. The authors designed mRNA injection protocols and transgenic systems to provide biotin directly in the egg water and found that a 12-hour labeling time was sufficient for biotinylation of the target proteins. The LMNA-adjacent molecules detected in both systems were enriched in the nuclear envelope and nuclear membrane proteins, including homologs of many lamin A-adjacent protein molecules identified in mammalian cells [117]. In addition, Xiong et al. developed a novel transgenic TurboID-dGBP zebrafish cell line using the proximity labeling technique. This cell line fused TurboID to a conditionally stable GFP-binding nanobody (dGBP). The dGBP directed TurboID to GFP-labeled target proteins, and the TurboID-dGBP zebrafish line allowed in vivo proximity labeling in live zebrafish by crossing with existing GFP-labeled zebrafish lines. This technique not only avoided the need to create transgenic organisms by fusing the protein of interest (POI) with the biotin ligase but also combined TurboID with dGBP to achieve GFP-directed proteome mapping using a modular system [118].

The Apaf-1-Caspase-9 complex, also known as the apoptosome, is a conserved cell death platform in multicellular organisms, and caspases are enzymes involved in programmed cell death. Recent studies have shown that caspases also play a crucial role as essential enzymes in many nonlethal cellular processes, referred to as caspase-dependent nonlethal cellular processes (CDPs). In 2019, Shinoda et al. labeled TurboID at the C-terminus of caspases, which not only revealed differences among proteins adjacent to caspases but also revealed the important role of the cleavage of the caspase substrate Acinus in Drosophila wing growth [106]. In 2021, Zhang et al. first labeled TurboID or miniTurbo at the C-terminus of CTP synthase (CTPS) and then detected conformational changes in the CTPS filamentous structure. They found that miniTurbo disrupted the normal structure of the CTPS cytoskeleton in Drosophila cells. The authors applied TurboID to various developmental stages and tissues of Drosophila and found that TurboID could label multiple developmental stages and obtain proteins near CTPS in various tissues. Furthermore, they discovered that TurboID-mediated biotinylation in Drosophila was driven by cell-specific Gal4 drivers [103]. Uçkun et al. introduced TurboID and miniTurbo into the endogenous anaplastic lymphoma kinase (Alk) locus of Drosophila through CRISPR/Cas9-mediated genome editing and used LC‒MS3 technology to identify a broad range of neuronal Alk proximal interactors and potential components of Alk signaling complexes. They further revealed that Stardust (Sdt), Discs large 1 (Dlg1), and others were coexpressed with Alk in the central nervous system and identified the protein-tyrosine-phosphatase Corkscrew (Csw) as a regulator of Alk signaling [119].

In 2022, Rayaprolu et al. used mice with specific TurboID expression to compare the CAMK2A-neuron and ALDH1L1-astrocyte proteomes. By doing so, they not only identified differences in brain-region-specific proteomes between the two cell types but also revealed distinctions in signaling phosphoproteins and cytokines derived from neurons and astrocytes [120]. Sun et al. constructed recombinant adeno-associated viruses (AAV) to express TurboID driven by cell-type-specific promoters. They intravenously injected these viruses into mouse brains and combined biotin affinity purification with microgram-scale TMT–LC/LC‒MS/MS to perform in-depth analysis of over 10,000 proteins from neurons or astrocytes. They also confirmed that TurboID could label various cell proteins in human HEK293 cells [121]. In 2023, Sunna et al. used stable TurboID-expressing mouse neuroblastoma (N2A) and microglial (BV2) cell lines, followed by biotinylated capture, to obtain 65% of N2A and 59% of BV2 proteomes. Protein analysis by MS revealed effects after lipopolysaccharide treatment (> 500 differentially abundant proteins), including increased abundance of typical proinflammatory proteins (Irg1 and Oasl1) and decreased abundance of anti-inflammatory proteins (Arg1 and Mgl2) [122].

#### Application in plants

In recent years, researchers found that TurboID not only functions effectively in animal models but also has wide applications in plants [123]. However, its application in plants has been slower due to the structural features of plants, such as the cell wall and cuticle, as well as the growth temperature selection and the ability of plants to produce and store biotin in cells. These characteristics limit the labeling of transient proteins and result in lower biotinylation efficiency in plants [124–126]. Although TurboID is the same size as BioID, its 14 amino acid mutations significantly enhance the labeling efficiency, making it a more suitable option for plant applications. Compared to BioID and BioID2, TurboID exhibits faster labeling kinetics and does not require high-temperature catalysis or similar restrictions, making it advantageous for use in plants. Notably, however, TurboID is the most promiscuous biotin ligase (PBL), which may lead to a reduced signal-to-noise ratio in experiments conducted in plants [127, 128].

In 2020, Arora et al. applied TurboID to *Lotus japonicus* symbiotically active receptor kinases. They not only identified known and unknown interacting endocytic TPLATE complexes but also demonstrated the superiority of TurboID in capturing membrane-associated protein interactions. Through experiments, the authors found that in tomatoes, the PBL-mediated biotin labeling efficiency increased with the addition of biotin, and the proximity labeling efficiency depended on the growth temperature. PBL was shown to promote cis-biotinylation of *Streptococcus pyogenes*. TurboID aided in capturing the periplasmic interactions of *S. pyogenes*, which provided new insights into identifying transient signaling components, new plant immune regulatory factors, and the efficient capture of cell and subcellular compartment-specific interactomes [126].

In 2021, Zhang et al. found that TurboID is more efficient than BioID in labeling proteins near the target protein in plants. They used the N-terminal Toll/interleukin-1 receptor (TIR) domain of the nucleotide-binding leucine-rich repeat (NLR) protein family as a model to identify protein interaction partners step by step. The method involved vector construction, agroinfiltration of protein expression constructs in plants, biotin treatment, protein extraction and desalting, quantification, and affinity purification of biotinylated proteins. The results demonstrated that TurboID can be used to study certain proteins in the tobacco genus and other plant species [86]. Since TurboID is relatively large compared to GFP (35 kDa), its fusion with the target protein may affect the functionality of the target protein. In such cases, a smaller version called miniTurbo can be used [62]. In 2023, Kim et al. used the TurboID proximity labeling technique to selectively capture kinases and phosphatase substrates. They combined this with mass spectrometry analysis and identified over 400 proximal proteins of *A. thaliana* BRASSINOSTEROID-INSENSITIVE2 (BIN2). Most of the proximal proteins showed BIN2-dependent phosphorylation in vivo or in vitro, indicating that they are substrates of BIN2. Additionally, the authors established the BIN2 signaling network through proteome analysis and revealed the role of BIN2 in regulating key cellular processes, such as transcription, RNA processing, translation initiation, vesicular trafficking, and cytoskeletal organization [78].

Nucleotide-binding leucine-rich repeat (NLR) immune receptors play a crucial role in defending against pathogens in both plants and animals [129, 130]. However, there is currently limited research on the mechanisms of NLR protein interactions and the regulation of NLR levels. In 2019, Zhang et al. used TurboID to identify the N-terminal protein interactome of a *tobacco mosaic virus* (TMV)-resistant NLR. Subsequently, through proteomic analysis and genetic screening, they revealed various regulatory factors involved in N-mediated immunity. They also found that the E3 ubiquitin ligase ubiquitin protein ligase E3 component N-recognin 7 (UBR7) directly interacts with the TIR domain of N, and the downregulation of UBR7 leads to increased N protein levels and enhanced TMV resistance. These findings demonstrate the role of TurboID-based proximity labeling in plants [33].

Elucidating the enzyme-substrate relationships in the posttranslational modification (PTM) network is crucial for understanding signal transduction pathways. However, the interactions between enzymes and substrates are often transient. Therefore, in 2019, Andrea used TurboID to study the proximity labeling of protein complexes and cell-type-specific proteomes in Arabidopsis, demonstrating that TurboID and miniTurbo can selectively study protein interactions in plants and explore localized protein interaction networks, identifying rare proteins or specific cell types [128]. Since TurboID and miniTurbo are derived from yeast, their appropriate operating temperature is room temperature or slightly above room temperature. TurboID and miniTurbo are inactive when at low temperatures, which may limit their application in plants (e.g., cold adaptation and cold stress experiments).

Researchers also compared the differences between TurboID and miniTurbo, two biotin ligases, in parameters such as temperature, incubation time, and biotin dosage. To demonstrate the applicability of TurboID and miniTurbo in plants, they were expressed in both peppermint and Arabidopsis and induced protein biotinylation with biotin treatment. The results showed that self-labeling could be achieved within 1 h after biotin treatment, indicating that TurboID and miniTurbo exhibited significantly higher activity than BirA. This result is consistent with observations in other organisms. Furthermore, as plants produce and store endogenous biotin in cells, background labeling may occur without exogenous biotin. However, in most cases, this background labeling can be ignored. When TurboID and miniTurbo were directly compared in peppermint, they showed similar activity and background labeling. However, in Arabidopsis, TurboID not only exhibited higher activity than miniTurbo but also generated more background labeling. The enhanced activity of TurboID was particularly evident in the low-level expression spectra of TurboID and miniTurbo in the absence of exogenous biotin. In 2023, Feng et al. used TurboID proximity labeling technology to study meiotic cells in Arabidopsis. They fused TurboID to two meiotic chromosome axis proteins, ASYNAPTIC 1 (ASY1) and ASYNAPTIC 3 (ASY3), and identified 39 neighboring proteins of ASY1 and/or ASY3 through affinity purification and mass spectrometry. This included most known chromosome axis-related proteins and newly discovered meiotic proteins. The study confirmed that TurboID-based proximity labeling in meiotic cells can identify proteins near the chromosome axis in Arabidopsis [131].

The formation of hairy roots is mediated by the expression of T-DNA-encoded genes from the root-inducing (Ri) plasmid, including the root oncogenic locus B (RolB) gene. RolB plays a major role in hairy root development, but the exact molecular function of the protein encoded by this gene is still unclear. In 2023, Gryffroy et al. applied TurboID proximity labeling technology to hairy roots in tomato (*Solanum lycopersicum L.*) and discovered new interacting partners that inhibit the proteins TOPLESS (TPL) and JAZ (NINJA), which directly interact with RolB. RolB can alleviate the function of TPL, leading to specific changes in plant hormone signaling, immunity, growth, and development processes, which provided important insights into the pathogenesis of hairy root disease (HRD) [132].

#### Applications in microbiology

Due to their significantly higher activity compared to other biotin ligases, TurboID and miniTurbo are not only suitable for mammalian cells and plants but also for microorganisms such as bacteria, yeast, viruses, and the nematode *Caenorhabditis elegans. Caenorhabditis elegans* is one of the most extensively studied multicellular eukaryotes in biology. In 2021, Sanchez et al. applied TurboID to live *Caenorhabditis elegans*. The authors constructed the TurboID plasmid and injected it into *Caenorhabditis elegans*, followed by experiments in biotin-rich or biotin-depleted bacterial dishes. TurboID was found to provide tissue- and region-specific promiscuous biotinylation in *Caenorhabditis elegans*. Additionally by studying non-centromeric microtubule organizing centers (ncMTOCs) in intestinal cells, it was demonstrated that its tissue-specific and region-specific proximity markers make it suitable for in vivo targeted protein network analysis [133]. Artan et al. optimized the proximity labeling approach using TurboID for *Caenorhabditis elegans*. The high affinity of biotin for streptavidin in TurboID allows biotin-labeled proteins to be affinity-purified under harsh denaturing conditions. By combining extensive sonication with denaturing agents such as SDS and urea, the authors achieved nearly complete solubilization of worm proteins. Subsequently, this method was used to characterize the proteomes of the *Caenorhabditis elegans* intestine, muscles, skin, and nervous system.

Neurons are among the smallest cells in *Caenorhabditis elegans*. The synaptic active zone consists of a protein matrix that is difficult to solubilize and purify. To validate whether the approach can solubilize proteins from the active zone, the authors introduced TurboID into the endogenous ELKS-1 gene, which encodes a presynaptic active zone protein. They identified numerous known active zone proteins interacting with ELKS-1, as well as previously unidentified synaptic proteins [134]. Due to the abundance of endogenous biotinylated proteins, especially carboxylases that use biotin as a cofactor (such as POD-2/acetyl-CoA carboxylase, PCCA-1/propionyl-CoA carboxylase, PYC-1/pyruvate carboxylase, and MCCC-1/methylcrotonyl-CoA carboxylase), which can reduce TurboID sensitivity, in 2022, the authors added a C-terminal His10 tag to these genes and subsequently removed them from the worm lysate using immobilized metal affinity chromatography. With this method, the authors improved the interactome of the presynaptic active zone protein ELKS-1, identifying many previously unknown potential synaptic proteins (such as human endothelin homolog F59C12.3). This method economically and efficiently addresses common contamination issues in proximity labeling and may be applicable to other model organisms, enabling a more in-depth and comprehensive analysis of interacting partners for proteins of interest [135]. In 2022, Holzer et al. used TurboID to identify tissue-specific centrosome components in *Caenorhabditis elegans* and successfully detected interactions between the stable-associated component SPD-5 and the dynamically localized component Polo-like kinase 1 (PLK-1). The authors further developed an indirect proximity labeling method using GFP nanobodies fused to TurboID, allowing the tissue-specific identification of protein interactomes throughout the entire animal. With this method, they identified homologs of two highly conserved centrosome components, centrosomal protein 97 (CEP97) and BLD10/CEP135, which are present in various somatic tissues of the worm [136]. Hertz et al. applied TurboID labeling and purification to localize proteins in the P granules of *Caenorhabditis elegans* embryos, revealing the proteome of worm P granules. This method can be used to study other membranous organelles in multicellular organisms [137].

Among other studies, TurboID has played a crucial role in exploring the nuclear pore complex (NPC) and mitochondrial proteins as potential drug targets in *Plasmodium*, the parasite responsible for malaria. In 2022, Ambekar et al. used TurboID to investigate orphan protein function in the malaria parasite and identified ten nucleoporins (Nups) that contribute to further research on NPC dynamics, structural elements, nucleocytoplasmic transport, and unique nontransport functions of nuclear pore proteins [79]. Currently, malaria infection and mortality rates remain high, partially due to the emergence of parasites resistant to frontline antimalarial drugs. *Plasmodium* is the deadliest species among human malaria parasites, and its mitochondrial function is the target for drugs such as atovaquone and proguanil (Malarone). Lamb et al. fused the mitochondrial targeting sequence of the Hsp60 molecular chaperone with TurboID and identified 122 putative mitochondrial proteins. To validate the mitochondrial localization of these proteins, they targeted four functionally uncharacterized candidates to the mitochondria and confirmed that three of them are indeed essential mitochondrial proteins. This research not only enhances our understanding of the mitochondrial proteome in *Plasmodium* but also enriches basic mitochondrial biology studies [138].

TurboID also plays an important role in research on *Toxoplasma gondii*, filamentous fungi, *Trypanosoma cruzi*, and *Chlamydomonas reinhardtii*. In *Toxoplasma gondii*, surface antigen 1 (TgSAG1) is a surface protein of the tachyzoite stage and plays a crucial role in parasite infection and host cell immune modulation. However, the mechanisms through which TgSAG1 regulates these processes remain unclear. In 2021, Zhou et al. fused TurboID with TgSAG1 and identified host proteins interacting with TgSAG1. The authors found that when Toxoplasma attaches to host cells, S100A6 colocalizes with TgSAG1. Disrupting or blocking S100A6’s function at its binding site inhibits parasite invasion. Additionally, TgSAG1 can inhibit the interaction between host cell vimentin and S100A6, promoting cytoskeletal reorganization during parasite invasion [132]. In 2022, Hollstein et al. demonstrated the application of TurboID in the filamentous fungus *Sordaria macrospora* (Sm) using one subunit of the striatin-interacting phosphatase and kinase complex (SmSTRIPAK). They fused codon-optimized TurboID biotin ligase with SmSTRIPAK complex interactor 1 (SCI1) and identified known SmSTRIPAK components (PRO11, SmMOB3, PRO22, and SmPP2Ac1) through affinity purification and mass spectrometry, indicating its successful application in filamentous fungi [139].

*Trypanosoma cruzi* is a flagellated protozoan closely related to human Chagas disease. It commonly infects invertebrates and mammals, utilizing its single flagellum for locomotion and establishing intimate interactions with hosts under specific conditions. Currently, the functions of the flagellum in *Trypanosoma cruzi*, apart from its role in locomotion, remain unclear and lack proteomic research. In 2023, Madalyn M. Won et al. utilized TurboID to target different compartments of the flagellum and cytoplasm during the replicative stage of *Trypanosoma cruzi*. Using mass spectrometry, they identified 218 flagellum-enriched proteins. Among them, 40 flagellum-enriched proteins were found to occur in all life stages of two parasitic organisms, including homologs of known flagellar proteins from other trypanosome species and proteins specific to the *Trypanosoma cruzi* lineage [140]. Studies have shown that phase separation is involved in many important cellular processes such as RNA metabolism, signal transduction, and stress granule assembly. However, organelles formed through phase separation are highly sensitive to environmental conditions, making them difficult to study using traditional proteomic techniques such as affinity purification-mass spectrometry. In *Chlamydomonas reinhardtii*, Rubisco is concentrated in a prominent phase-separated organelle known as the pyrenoid. In 2023, Chun Sing Lau et al. used TurboID to label proteins with biotin radicals, thereby marking proximal proteins in *Chlamydomonas reinhardtii* chloroplasts. The authors generated a high-confidence proximal proteome of the pyrenoid, which not only included most of the known pyrenoid proteins but also revealed numerous new candidate pyrenoid proteins [141].

The global pandemic caused by the novel coronavirus (SARS-CoV-2) has led to infections in over 200 million people [142]. To expand our understanding of the interactions between SARS-CoV-2 and humans, in 2020, V’kovski et al. used three proximity labeling methods, BioID, TurboID, and APEX2, to determine the molecular microenvironment of the coronavirus replication/transcription complex (RTC), i.e., the proteins located around the RTC. These factors represent the molecular characteristics of the coronavirus RTC and provide important insights for antiviral intervention strategies [143]. In 2021, Chen et al. published a study in which they fused 29 viral proteins to either BioID2 or the S protein-FLAG-streptavidin-binding peptide (SFB) tag and performed proximity labeling and tandem affinity purification (PL-TAP). These interacting partners confirmed previous research results and revealed additional interactors of SARS-CoV-2 proteins, which could be potential drug targets. To further elucidate the molecular mechanisms between the virus and the host, in 2022, Shang et al. employed an antibody-based TurboID proximity labeling approach to screen for molecular interactions of SARS-CoV-2 proteins. This technique directly identifies the biotinylated peptides of TurboID-labeled viral proteins, aiding in the identification of 1388 high-confidence proximal interactors of SARS-CoV-2 proteins, 1092 of which were not covered by the chain-avidin-based BioID in previous interactome studies [144]. Zhang et al., using TurboID, investigated the interactions of 29 viral proteins in human cells and found that SARS-CoV-2 manipulates antiviral and immune responses. SARS-CoV-2 proteins inhibit the activation of the interferon pathway through mitochondrial antiviral-signaling protein (MAVS), SET domain-containing 2 (SETD2), and histone lysine methyltransferase SETD2. The authors proposed 111 potential drugs for the clinical treatment of COVID-19 (coronavirus disease 2019) and identified three compounds that significantly inhibit SARS-CoV-2 replication, laying a foundation for understanding the viral infection mechanism and developing therapeutic drugs for COVID-19 [145].

## Summary and outlook

In summary, this article provides a detailed introduction to the development and principles of proximity labeling techniques and summarizes the applications of various proximity labeling enzymes in mammals, plants, and microorganisms in recent years. The development of proximity labeling enzymes such as APEX, BioID, and TurboID has opened up new avenues for small-molecule research and improved the study of protein-nucleic acid interaction networks, gradually becoming an indispensable part of cell biology, neurobiology, immunology, virology, and other fields.

Currently, this technology is widely used not only in determining the proteomic localization of mammalian protein complexes and cellular structures but also in exploring the proteome of plants in response to environmental signals, with unique advantages in studying proton pump inhibitors and low-abundance membrane-localized proteins. In addition, it has enriched the known proteomic networks of organisms such as *Caenorhabditis elegans*, Toxoplasma, *Phytophthora infestans*, and novel coronaviruses, making proximity labeling technology a powerful tool for biological research. Due to its efficiency, flexibility, and operability, combining proximity labeling with existing techniques can solve biological problems that are currently difficult to address, providing effective support for drug target discovery, cancer diagnosis, single-cell proteomics, and other research fields.

However, with the continual development of proteomics and proximity labeling technology, the limitations of this technology are increasingly prominent. For example, it cannot be used to detect nonadjacent interactions between proteins, high concentrations of biotin can label nontarget proteins in adjacent regions, and the pH differences between different subcellular compartments lead to different TurboID activities. Therefore, further optimization is still needed in the structural design and labeling experiments of APEX, BioID, and TurboID. Recently, researchers have combined APEX with RNA‒protein chemical crosslinking to create APEX-RIP and combined APEX2 with UV and RNA sequencing to invent Proximity-CLIP and APEX-seq. These findings open up new avenues for studying RNA‒protein interactions, RNA sequencing, the recognition of cis-regulatory elements, and their spatial localization. In the future, this technology can also be combined with gene editing or even laser editing techniques to achieve proximity labeling of target proteins and rapidly annotate the related physiological processes caused by protein interactions.

## Data Availability

Not applicable.

## Abbreviations

AAV: Adeno-associated viral
Ago2: Argonaute 2
Alk: Anaplastic lymphoma kinase
APEX: Ascorbate peroxidase
APEX-MS: APEX mass spectrometry
Arg: Arginine
ASY1: ASYNAPTIC 1
ASY3: ASYNAPTIC 3
ATG8: AuTophaGy-related 8
ATG9A: Autophagy-related protein 9A
ATG13: Autophagy-related protein 13
ATGs: Autophagy-related proteins
BiFC: Bimolecular fluorescence complementation assay
BIN2: BRASSINOSTEROID-INSENSITIVE2
BirA: an *Escherichia coli *biotin protein ligase mutant
BP: Biotin-phenol
Btn-An: Biotin-phenylamine
BV2: Mouse microglial
CDPs: Caspase-dependent nonlethal cellular processes
CEP97: Centrosomal protein 97
ChIP-seq: Chromatin immunoprecipitation sequencing
Co-IP: Co-immunoprecipitation
COVID-19: Coronavirus disease 2019
CRIP2: Cysteine-rich protein 2
CRL4CRBN: CUL4-DDB1-CRBN-RBX1
Csw: Corkscrew
CTPS: CTP synthase
CUL4: Cullin 4
DAB: Detect diaminobenzidine
dGBP: Conditionally stable GFP-binding nanobody
Dlg1: Discs large 1
EARP: Endosome-associated recycling protein
eCRs: Engineered chromatin readers
EM: Electron microscopy
EndoU: Endoribonuclease
ER: Endoplasmic reticulum
ErbB2: Human epidermal growth factor receptor 2
ERM: Ezrin-radixin-moesin
EV: Empty vector
FACS: Fluorescence-activated cell sorting
FL-Bid: Full length BH3-interacting domain death agonist
FLYWCH1: FLYWCH/Zn-finger DNA-binding domain
FKBP: FK506-binding protein
FRB: FKBP12-rapamycin-binding
GARP: Glycoprotein A repetitions predominant
Gly: Glycine
GIGYF2: Grb10-interacting GYF protein 2
GST Pull-down Assay: Glutathione S-transferase pull-down assay
H3K4me3: Trimethylation of lysine 4 on histone H3
H3K9me3: Trimethylated histone 3 lysine 9
H3K27me3: Tri-methylation at lysine 27 of histone H3
HopF2: HopF2bPt0DC3000
HRD: Hairy root disease
HRP: Horseradish peroxidase
hTR: Human telomerase RNA
IKZF1: IKAROS Family Zinc Finger 1
IMC: Inner membrane complex
IMS: Intermembrane space
iPSD: Inhibitory postsynaptic density
LC-MS/MS: Liquid chromatography-tandem mass spectrometry
LMNA: Lamin A
MAVS: Mitochondrial antiviral-signaling protein
MDM2: Mouse double minute 2
miniSOG: Mini singlet Oxygen Generator
MP: Myristoylation/palmitoylation
N2A: Mouse neuroblastoma
ncMTOCs: Non-centrosomal microtubule-organizing centers
NINJA: Novel INteractor of JAZ
NLR: Nucleotide-binding leucine-rich repeat
NPC: Nuclear pore complex
NrCAM: Neuronal Cell Adhesion Molecule
Nrg1: Neuregulin 1
Nups: Nucleoporins
OMM: Outer Mitochondrial Membrane
ORF3a: Open-reading frames 3a
PBL: Promiscuous biotin ligase
PL: Proximity labeling
PLK-1: Polo-like kinase 1
PL-TAP: Proximity labeling and tandem affinity purification
PML: Promyelocytic leukemia
PML NBs: PROMYELOCYTIC Leukemia Nuclear Bodies
POI: Protein of interest
PPIs: Protein-protein interactions
Pst: *Pseudomonas syringae* pv. tomato
PTM: Posttranslational modification
PUP-IT: Pupylation-based interaction tagging
RBP: RNA binding protein
RBX1: RING-box protein 1
ReAsH: Resorufin-based label
Ri: Root-inducing
RIP-seq: RNA immunoprecipitation sequencing
RolB: Root oncogenic locus B
ROS: Reactive oxygen species
RTC: Replication/transcription complex
SAINT: Significance analysis of INTeractome
SALL4: Spalt-like transcription factor 4
sAPEX: split-APEX
SARS-CoV-2: Severe acute respiratory syndrome coronavirus 2
SCI1: STRIPAK complex 1
Sdt: Stardust
SETD2: SET domain-containing 2
SFB: S protein-FLAG-streptavidin-binding peptide
Sm: Sordaria macrospora
SmSTRIPAK: Sordaria macrospora using the striatin-interacting phosphatase and kinase complex
STRIPAK: Striatin-interacting phosphatase and kinase
SUMO: Small Ubiquitin-like Modifier
TbMORN1: T. brucei MORN1
TCR: T-cell receptor
TgSAG1: Toxoplasma gondii, the surface antigen 1
TIR: Toll/interleukin-1 receptor
TMV: *Tobacco mosaic virus*
TPL: TOPLESS
TRAPP: TRAnsport Protein Particle
TRE: Tetracycline response elements
TSA: Tyramide Signal Amplification
tTA: Tetracycline-controlled transcriptional activator
UBR7: Ubiquitin protein ligase E3 component N-recognin 7
ULK1: UNC-52-like kinase 1
UPD: Upstream pseudo-knot domain
UV: Ultraviolet
VDAC2: Voltage-dependent anion-selective channel protein 2
