# Correction: The development of proximity labeling technology and its applications in mammals, plants, and microorganisms

**DOI:** 10.1186/s12964-024-01750-3

**Published:** 2024-08-16

**Authors:** Jieyu Guo, Shuang Guo, Siao Lu, Jun Gong, Long Wang, Liqiong Ding, Qingjie Chen, Wu Liu

**Affiliations:** 1 School of Basic Medical Sciences, Xianning Medical College, Hubei University of Science and Technology, Xianning, Hubei 437000 China; 2 School of Pharmacy, Xianning Medical College, Hubei University of Science and Technology, Xianning, Hubei 437000 China; 3 Medicine Research Institute, Hubei Key Laboratory of Diabetes and Angiopathy, Xianning Medical College, Hubei University of Science and Technology, Xianning, Hubei 437000 China

**Correction: Cell Commun Signal 21, 269 (2023)**

10.1186/s12964-023-01310-1

Following publication of the original article [1], it was reported that there was an error in reference 139.

The correct reference is:

Hollstein LS, Schmitt K, Valerius O, Stahlhut G, Pöggeler S: Establishment of in vivo proximity labeling with biotin using TurboID in the filamentous fungus Sordaria macrospora. Sci Rep 2022, 12:17727.

In the section, Applications in microbiology, the authors would like to correct the text in the fourth paragraph, from:

In 2022, Hollstein et al. demonstrated the application of TurboID in the filamentous fungus *Sordaria macrospora* (Sm) using the striatin-interacting phosphatase and kinase complex (SmSTRIPAK). They fused codon-optimized TurboID biotin ligase with striatin-interacting phosphatase and kinase (STRIPAK) complex 1 (SCI1) and identified known SmSTRIPAK components (PRO11, SmMOB3, PRO22, and SmPP2Ac1) through affinity purification and mass spectrometry, indicating the successful application of TurboID in filamentous fungi [139].

To:

In 2022, Hollstein et al. demonstrated the application of TurboID in the filamentous fungus *Sordaria macrospora* (Sm) using one subunit of the striatin-interacting phosphatase and kinase complex (SmSTRIPAK). They fused codon-optimized TurboID biotin ligase with SmSTRIPAK complex interactor 1 (SCI1) and identified known SmSTRIPAK components (PRO11, SmMOB3, PRO22, and SmPP2Ac1) through affinity purification and mass spectrometry, indicating its successful application in filamentous fungi [139].

The original article [1] has been corrected.
